# Moderate Adolescent Ethanol Vapor Exposure and Acute Stress in Adulthood: Sex-Dependent Effects on Social Behavior and Ethanol Intake in Sprague–Dawley Rats

**DOI:** 10.3390/brainsci10110829

**Published:** 2020-11-07

**Authors:** Meredith E. Gamble, Marvin R. Diaz

**Affiliations:** 1 Department of Psychology, Binghamton University, Binghamton, NY 13902, USA; mgamble1@binghamton.edu; 2 Center for Development and Behavioral Neuroscience, Binghamton University, Binghamton, NY 13902, USA; 3 Developmental Exposure Alcohol Research Center, Binghamton University, Binghamton, NY 13902, USA

**Keywords:** adolescent ethanol exposure, stress, alcohol, anxiety, ethanol intake, social

## Abstract

Adolescent alcohol use can lead to numerous consequences, including altered stress reactivity and higher risk for later anxiety and alcohol use disorders. Many studies have examined the consequences of heavy ethanol exposure in adolescence, but far less is understood about lower levels of intoxication. The present study examined moderate adolescent ethanol exposure as a possible factor in increasing stress reactivity in adulthood, measured through general and social anxiety-like behaviors, as well voluntary ethanol intake. Male and female Sprague–Dawley rats underwent an adolescent chronic intermittent ethanol (aCIE) vapor exposure during early adolescence, reaching moderate blood ethanol concentrations. Animals then underwent two days of forced swim stress in adulthood. We found that ethanol-exposed males consumed more ethanol than their air counterparts and an interesting stress and ethanol exposure interaction in males. There were no significant effects on voluntary drinking in females. However, the social interaction test revealed increased play-fighting behavior in ethanol-exposed females and reduced social preference in females after two days of stress exposure. Overall, this work provides evidence for sex-specific, long-term effects of moderate aCIE and susceptibility to acute stress in adulthood.

## 1. Introduction

Adolescence is a period of extensive neurodevelopmental and behavioral changes and has been associated with heightened levels of risk-taking and reward-seeking behaviors, including problematic alcohol use [1,2]. As of 2018, 7.4 million individuals under the age of twenty-one reported drinking in the past month [3]. In the United States, young adolescence, particularly between the ages of twelve and fourteen years old, has been identified as a critical age for the initiation of and rapid increase in use of alcohol, with more than ten percent meeting criteria for alcohol use disorder (AUD) within the first year of initiation [4]. This early initiation of alcohol use is strongly associated with increased alcohol drinking and heightened risk for developing AUD [5,6]. Adolescent alcohol use has been linked with many negative consequences and neural alterations that can last well into adulthood (for review, see [7,8]). Additionally, adolescent alcohol use has been linked with higher rates of other forms of psychopathology, including anxiety and depression [9,10,11,12].

Studies examining adolescent ethanol exposure in animal models have also shown many long-term behavioral impacts, including alterations to anxiety-like behaviors and voluntary ethanol intake in adulthood. For example, adolescent intermittent ethanol (AIE) exposure via intraperitoneal injection (2–3 g/kg; blood ethanol concentrations [BECs] ~185–250 mg/dL) in rats increased anxiety-like behaviors using both the elevated plus-maze and light–dark box, as well as increased voluntary ethanol intake in adulthood [13,14,15,16]. Additionally, males that underwent AIE (intragastric gavage; 3.5 and 4 g/kg BECs ~130–200 mg/dL) during early adolescence show higher levels of social anxiety-like behavior in adulthood, an effect not seen in females [17,18]. In contrast, others have found no change in anxiety-like behavior in the elevated plus-maze when adolescent ethanol was administered via intragastric gavage (3 g/kg; BECs ~150–180 mg/dL) [19] and no change in ethanol intake escalation [20], along with decreased anxiety-like behavior in the elevated plus-maze following AIE vapor exposure (14 h a day, two days on and two days off, average BECs > 300 mg/dL) [21]. A similar decrease in anxiety-like behavior was observed using another adolescent vapor exposure paradigm (five two-hour exposures, BECs ~172 mg/dL) in both the light–dark box and elevated plus-maze [22]. Interestingly, sex differences have been repeatedly reported, with males showing more long-term deficits, especially relating to social anxiety-like behaviors, while females may be more vulnerable to increased ethanol intake in adulthood following adolescent ethanol exposure [17,23,24,25,26]. Together, these studies suggest that the effects of adolescent ethanol exposure are quite variable, particularly at relatively high levels of ethanol exposure.

Importantly, though, while these long-term effects following high-level ethanol exposure in adolescence have been extensively examined, with many studies using methods that yield BECs far exceeding the defined level for human binge drinking at 80 mg/dL [27], the impacts of lower levels of exposure are far less understood. This poses a major problem for translatability, as not all human adolescents routinely drink to these levels [28]. In humans, moderate drinking is defined as no more than one drink per day for women and no more than two per day for men. This typically results in blood alcohol levels less than 80 mg/dL [29]. As of 2018, of the adolescents aged twelve to twenty that reported drinking in the past month (18.8%), approximately 5.1% did not indicate binge or heavy use [28]. Importantly, though, moderate drinking does increase risk for several significant health consequences [30]; however, how moderate drinking may contribute to AUD and other neurobehavioral disorders later on has not been extensively examined. Nevertheless, of the few preclinical studies examining lower levels of exposure, one used intermittent voluntary consumption of 10% sweetened ethanol in adolescence and demonstrated increased sweetened ethanol intake in adulthood; however, as is typical of voluntary drinking models, the levels of intoxication were highly variable [31]. In another study using a voluntary drinking model with unsweetened 20% ethanol during adolescence, average BECs were relatively low (~22 mg/dL) but results still showed increased drinking in adulthood [32]. While these results are consistent with those following ethanol exposures that produce substantially higher BECs, consequences of moderate ethanol exposures are highly under-represented, emphasizing the need for continued research examining the effects of lower levels of intoxication, which are still relevant to the adolescent population. Furthermore, there are additional factors that may contribute to, or interact with, ethanol-induced alterations to behavior, including exposure to stress.

Stress exposure is strongly associated with both anxiety and alcohol intake, and it has been suggested that stressful events may act as moderators that activate or amplify underlying vulnerability for different psychopathologies, such as anxiety and depression (for review, see [33]). Individuals under stressful conditions tend to gravitate towards the extremes of alcohol use; they may abstain from alcohol use altogether or engage in problematic drinking behavior [34]. Additionally, both human and animal studies have shown that females tend to be more vulnerable to stress-reactive drinking [35,36]. Importantly, an individual’s vulnerability to acute stress in adulthood may be affected by alcohol misuse during adolescence, as there is continued maturation of the major physiological stress response system, the hypothalamic–pituitary–adrenal (HPA) axis (for review, see [37]) through adolescence. For example, following AIE (intragastric; 4 g/kg), basal corticosterone (CORT) levels were elevated [38] and overall HPA axis reactivity was increased [39] in adult females. However, AIE-exposed males (intragastric; 4 g/kg) showed blunted CORT levels and vasopressin expression in the paraventricular nucleus in response to an ethanol challenge in adulthood [40]. This dysregulation to the stress system may manifest as behavioral alterations, including increased stress-reactive drinking in AIE adults following both forced swim stress (FSS) and foot shock stress [41,42] and decreased social interaction in adult AIE males one week following acute restraint stress [26]. Overall, these results provide evidence of stress-related neural circuitry dysregulation and subsequent behavioral alterations resulting from adolescent exposure to higher doses of ethanol. However, whether a more moderate exposure to alcohol during adolescence, which commonly occurs in humans, can heighten sensitivity to stress exposure in adulthood is not well understood.

The goal of the current study was to examine the impact of a more moderate adolescent ethanol exposure (as reviewed by [43]) on adult stress reactivity. To do this, male and female Sprague–Dawley rats underwent a moderate adolescent chronic intermittent ethanol (aCIE) vapor exposure and were tested on a variety of behavioral assays following exposure to FSS in adulthood. Rats were assessed for both general and social anxiety-like behavior, as well as voluntary ethanol intake.

## 2. Methods

### 2.1. Animals

Male and female Sprague–Dawley rats (*N* = 139 rats) were bred in house, with breeding pairs originating from Envigo (Indianapolis, IN, USA). Litters were culled to twelve animals, and at weaning (postnatal (P) 21) same-sex animals were housed two to three per cage and randomly assigned to an experimental condition. Rats continued to receive food and water ad libitum and were maintained on a 12 h light-dark cycle (lights on at 07:30). Rats began alcohol exposure in adolescence (P29–30) and were behaviorally tested in adulthood (P70+). All rats were treated following guidelines for animal care under protocols approved by the Binghamton University Institutional Animal Care and Use Committee (code: 789-17).

### 2.2. Experimental Design

**Figure 1:**
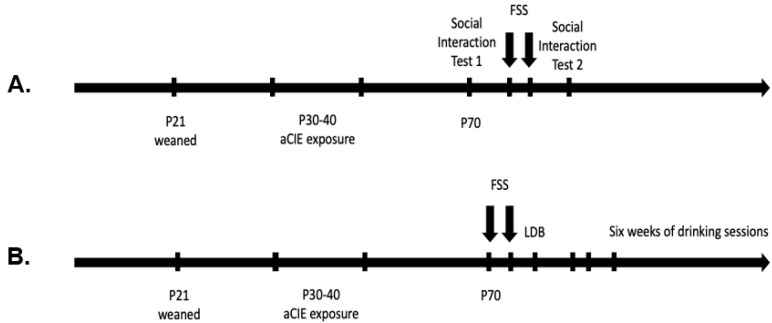
Experimental timelines, starting with animals weaned on postnatal day (P) 21, and the adolescent chronic intermittent ethanol exposure from P30–40. Starting on ~P70, animals that were tested for social interaction underwent the first test session on P70, followed by two days of FSS and then a second test session 24 h later (**A**). For experiment 2, assigned animals underwent two days of forced swim stress (FSS) followed by the light-dark box (LDB) and six weeks of voluntary drinking sessions, beginning the following Monday (**B**).

The experimental design was a 2 (adolescent exposure: ethanol vapor or air) × 2 (stressor exposure: stressed or non-stressed) factorial for each sex. At weaning, animals were randomly assigned to one of four groups (per sex): air/non-stressed, air/stressed, ethanol/non-stressed, or ethanol/stressed. All cage mates were assigned to the same condition. Rats went through a ten-day adolescent vapor exposure starting on P29–30, and then were left undisturbed until adulthood (~P70), when they began FSS sessions and their respective behavioral testing (Figure 1).

### 2.3. Moderate Adolescent Chronic Intermittent Ethanol (aCIE) Exposure

Starting on P29–30, rats were transferred to vapor inhalation chambers, as previously described [44], with two to five rats per cage; rats continued to receive food and water ad libitum, and food was changed immediately after each ethanol exposure. Weights were monitored throughout the exposure period. The aCIE exposure consisted of exposure to vaporized ethanol or room air every other night for 12 h (20:00–08:00), for a total of five exposures over ten days. Ethanol was replaced for each exposure day to prevent dilution throughout exposure period. Following the final exposure, rats were returned to original housing groups with cage mates assigned to the same adolescent exposure condition (two to three per cage) and returned to the colony room to age until adulthood (P70).

### 2.4. Blood Ethanol Concentrations

BECs were sampled in a separate group of aCIE animals (*n* = 8 per group, per sex, *N* = 32) to identify a time course of blood ethanol levels during the vapor exposures. Every two hours, cages were quickly removed from chambers, chamber door was closed, blood samples were collected, and then cages were again quickly returned to chambers until the next time point. This was true only for BEC data collection and did not occur during aCIE exposures for animals that were tested in adulthood. Tail blood (~100 µL) was collected and centrifuged, serum was extracted, and samples were stored at −20 °C. BECs were determined using an Analox AM-1 alcohol analyzer (Analox Instruments, Lunenburg, MA, USA). The samples were run in duplicate with 5 µL aliquots, and the order of samples was randomized. The machine was calibrated at 100 mg/dL, and a Quality Control known concentration of ethanol was assessed between sets of samples to ensure accuracy throughout.

### 2.5. Forced Swim Stress

Once rats reached adulthood (P70+), the stressed groups underwent two days of FSS, as previously reported [45]. All cage mates were assigned to the same stressor condition. On the day of the exposure, experimental subjects were transported to testing room between 09:00 and 11:00 h and placed simultaneously in individual polycarbonate cylinders (height = 45.72 cm, diameter = 20.32 cm) filled with water to a depth of 25 cm at 25 °C for ten minutes on two consecutive days, 24 h apart. During the ten-minute testing session, the behavior of the rats was recorded by video camera for later scoring and analysis. After each FSS exposure, rats were dried with a towel and placed in a new cage for 30 min to allow for drying time and were then returned to their home cage and colony. Rats assigned to a non-stressed condition were left un-manipulated in their home cage.

FSS videos were later scored by a trained experimenter that was blind to the experimental conditions of each animal. Of all the animals that underwent FSS, we randomly selected *n* = 8–9 per group, per sex for scoring. Ten-minute sessions were scored in five-second bins for three behaviors: climbing (animal is making active movement, typically against the wall of the cylinder, with front paws out of the water and propelling itself up so that the shoulders are above the water), swimming (animal is actively moving more than is necessary to keep its head above water), and immobility (animal is floating and only making the minimal movements required to keep its head above water) [46]. For each five second bin, the experimenter tallied which behavior was most prevalent, for a total of 120 time bins. The frequency of immobility, climbing, and swimming behaviors, as well as the latency to first immobile episode were scored for both testing sessions.

### 2.6. Experiment 1: Social Interaction

Testing was conducted, as previously described [45], under dim light (15–20 lx) in a Plexiglas (Binghamton Plate Glass, Binghamton, NY, USA) test apparatus (45 × 30 × 20 cm) containing clean bedding and divided into two equal-sized compartments by a clear Plexiglas partition. The partition contained a small opening (9 × 7 cm) to allow for movement of the rats between the two compartments.

On the day of testing, experimental subjects (*n* = 8 per group, per sex, *N* = 32) were taken from their home cage and placed individually in the testing apparatus for a 30-min habituation period; all testing procedures were conducted between 09:00 and 11:00. A novel social partner of the same age and sex and minimal weight discrepancy (not exceeding 30 g, experimental animals always heavier than novel partners) was then introduced for a ten-minute testing period. Partners were always unfamiliar with both the testing apparatus and the experimental animal, were not socially deprived prior to the test, and were experimentally naïve.

During the ten-minute testing session, the behavior was recorded by video camera for scoring and analysis later. The experimental animal was differentiated from the novel partner by a colored vertical stripe on the back. Rats were then returned to their home cage. The following two days, all experimental animals underwent the two ten-minute FSS sessions (24 h apart). Subsequently, 24 h after the second session, the rats went through the social interaction testing again with another novel partner (Figure 1A).

The frequency of social investigation, contact behavior, and play fighting were scored from video recordings by a trained experimenter that was blind to the experimental conditions of each animal. Social investigation was defined as sniffing of any part of the body of the partner. Contact behavior was defined as crawling over and under the partner, as well as social grooming. Play fighting was scored as the sum of the frequencies of: pouncing at the partner (a playful nape attack where the experimental animal lunges at the partner with its forepaws extended outward), following and chasing of the partner (rapid pursuit of the partner by the experimental animal), and pinning (the experimental animal stands over the partner on its back, pressing it against the floor). No aggressive behavior was exhibited in these experiments; play fighting is distinguished from serious fighting by the target area of attack, where in play fighting, the target area is typically the nape of the partner. The final measure was the preference/avoidance coefficient, which is measured by the number of crossovers to the other compartment based on the location of the novel partner. The experimental animal either crosses over to the compartment with the novel partner (preference) or to the compartment away from the novel partner (avoidance). A coefficient of preference/avoidance was calculated as [coefficient percent = [(crossovers to the partner–crossovers away from the partner)/total crossovers) × 100].

### 2.7. Experiment 2: Light–Dark Box and Voluntary Ethanol Drinking

#### 2.7.1. Light–Dark Box

In a different group of rats, twenty-four hours after the second FSS session, animals (males: air non-stressed *n* = 10, air stressed *n* = 10, aCIE non-stressed *n* = 7, aCIE stressed *n* = 9; females: air non-stressed *n* = 10, air stressed *n* = 11, aCIE non-stressed *n* = 10, aCIE stressed n = 8; *N* = 75) went through the light–dark box (LDB) as an assessment for general anxiety-like behavior (Figure 1B). Non-stressed rats were handled two days prior to LDB testing to equate them to the handling of rats that underwent two days of FSS. The testing apparatus consisted of two attached chambers (34 × 24 ×24 cm), one made of white and one made of black opaque Plexiglas, with a small circular opening (8 × 8 cm) between the two, allowing for free movement of the animal between the two chambers. The white Plexiglas chamber was covered by a clear lid that allowed for room light to enter the chamber, whereas the black Plexiglas side was covered by a solid black lid that did not allow for any light to enter the chamber through it. The side made of white Plexiglas is deemed “the light side” and the side with black Plexiglas is the “dark side”. Testing took place under bright room lighting (280 lx), and the apparatus was cleaned with 3% hydrogen peroxide and dried completely before each animal was tested.

All testing occurred between 08:00 and 10:00. One cage at a time, cage mates were placed in individual cages and transported to the testing room. One at a time, rats were placed in the light side facing away from the aperture, and the researcher left the room for the five-minute test session. The test session was recorded by video camera for later analysis. After both cage mates completed their testing session, animals were returned to their home cage and colony room.

Videos were scored by a trained experimenter blind to the experimental condition of each animal. Animals were assessed for: total light time (the duration of time spent on the light side of the apparatus, with all four paws crossing through the aperture), egress latency (the duration to entering the dark side for the first time), transitions (the number of total crossovers, with all four paws through the aperture), and head pokes into the light side (the animal sticks only its head into the light side, with both ears crossing through the aperture).

#### 2.7.2. Voluntary Ethanol Drinking

The same rats were used after LDB for the drinking experiment. On the Monday following LDB testing (Thursday or Friday) animals began limited-access voluntary ethanol intake sessions every Monday, Wednesday, and Friday morning (starting between 08:00 and 08:30) for six weeks (a total of eighteen sessions) (Figure 1B). For each session, animals were transported to the testing room, weighed, and placed in individual cages with normal bedding. Rats were given free access to one bottle containing a sweetened ethanol solution (9.37% ethanol + 2.96% sucrose + 0.12% saccharin) for thirty minutes. Drinking bottles were weighed before and after testing to calculate g/kg of ethanol intake of each animal per session; daily intake averages per week and cumulative total intake over the six weeks were also calculated. After the drinking session, animals were returned to their home cage and colony room. Immediately after the final drinking session, tail blood samples (~100 µL) were collected to determine BECs of each animal and processed as described above.

### 2.8. Statistics and Data Analyses

Based on previous literature, sex differences have been well-established in all of the used behavioral measures [17,26], therefore, *a priori,* males and females were analyzed and reported independently for all experiments. Statistical analyses were performed using GraphPad Prism 8 (GraphPad, San Diego, CA, USA). BEC data were analyzed using a two-way ANOVA (hour × exposure day) and body weight data were analyzed using a two-way ANOVA (age × ethanol exposure) and a *t*-test to determine any differences at baseline. FSS data were analyzed using a two-way repeated-measures ANOVA with matching and Geisser–Greenhouse correction for variance (ethanol exposure × day). Social interaction data were analyzed using a two-way repeated measures ANOVA with matching and Geisser–Greenhouse correction for variance (ethanol exposure × stress exposure). LDB data were analyzed using a two-way ANOVA (ethanol exposure × stress exposure), and a Mann–Whitney non-parametric test was used for stress effects in male transitions. This was with the conservative assumption that these data were not normally distributed, as tests of normality could not be conducted with the low sample sizes. Total ethanol intake over the six weeks of drinking sessions was analyzed using a two-way ANOVA (ethanol exposure × stress exposure), and daily averages per week for ethanol intake were analyzed using a three-way repeated-measures ANOVA with matching and Geisser–Greenhouse correction for variance (ethanol exposure × stress exposure × week). In rare instances of missing ethanol intake data due to bottle spilling or leaking during intake session, the animal’s daily average for that week was used for that session; there were only about six instances of this throughout the entire experiment. Outliers were determined using GraphPad ROUT method (Q = 1%) and one female social interaction outlier was removed from all analyses. Sidak’s post-hoc test was used to follow up significant main effects and interactions.

## 3. Results

### 3.1. Characterization of the aCIE Model

**Figure 2:**
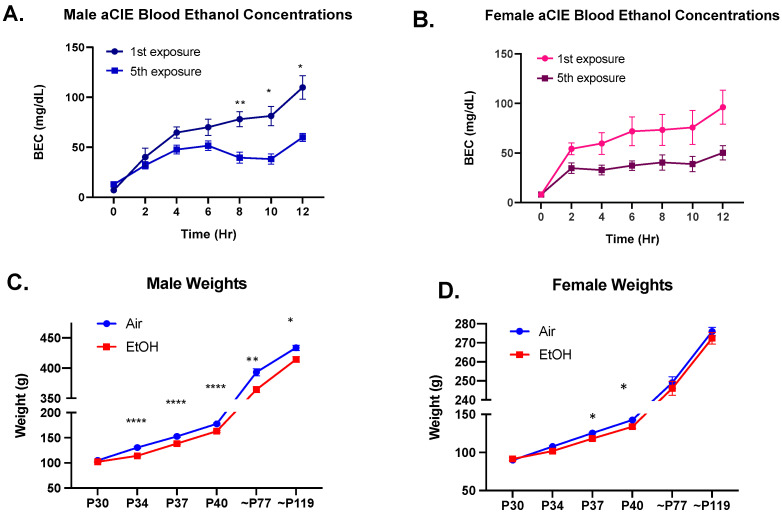
Blood ethanol concentration (BEC) time courses and weights. BECs over the twelve-hour adolescent chronic intermittent ethanol exposure for the first and the last exposure day for males (**A**) and females (**B**). Both males and females showed significantly lower BECs for the final exposure compared to first exposure; *n* = 8 per group, significant effect of exposure day denoted as * *p* < 0.05 and ** *p* < 0.01. Male (**C**) and female (**D**) weights throughout adolescent chronic intermittent ethanol exposure (P30-40) and on the first and last day of drinking sessions during adulthood (~P77 and~P119) compared between adolescent air or ethanol (EtOH) exposure. Ethanol-exposed males showed significantly lower weights throughout the aCIE exposure period and at both time points in adulthood, and ethanol-exposed females showed significantly lower weight on day seven and at the end of the aCIE exposure but did not show any differences in adulthood. A significant exposure effect denoted as: * *p* < 0.05, ** *p* < 0.01, and **** *p* < 0.0001.

Ethanol vapor concentrations in the chambers were taken at the end of each exposure and averaged at 4.1 ± 0.8 g/L. BEC time course was determined from a separate cohort of animals that underwent the same aCIE exposure. For males, this exposure yielded BECs that peaked at 109.8 ± 11.71 mg/dL during the first exposure and 59.94 ± 4.00 mg/dL during the final exposure, indicating a significant decrease in BECs between first and final exposure (Figure 2A; *F*(1,14) = 34.54, *p* < 0.0001); follow-up with Sidak’s post-hoc test revealed significantly lower BECs on the final exposure day for hours: eight (*p* = 0.008), ten (*p* = 0.016), and twelve (*p* = 0.023). For females, this exposure yielded BECs that peaked at 96.17 ± 17.16 mg/dL during the first exposure and 50.21 ± 7.18 mg/dL during the fifth exposure, also indicating a significant decrease in BECs between first and final exposure (Figure 2B; *F*(1,14) = 5.030, *p* = 0.042).

Body weights were recorded throughout the exposure period (on days four, seven, and ten) and before all drinking sessions in adulthood. Weights between air and ethanol-exposed animals did not significantly differ at the start of exposure (males: *t*(56.38) = 1.540, *p* = 0.129; females: *t*(51.54) = 0.888, *p* = 0.379); however, aCIE animals showed significantly lower weights throughout the exposure period and into adulthood compared to their air counterparts (males: *F*(1,66) = 25.76, *p* < 0.0001; females: *F*(1,69) = 5.007, *p* = 0.029). Interestingly, ethanol-exposed males continued to show this significant decrease in weight into adulthood on both the first and last day of drinking sessions (Figure 2C; first drinking session: *p* = 0.002; final drinking session: *p =* 0.022). Ethanol-exposed females did not show any significant differences in weight in adulthood at these same time points (Figure 2D).

### 3.2. Forced Swim Stress

**Figure 3:**
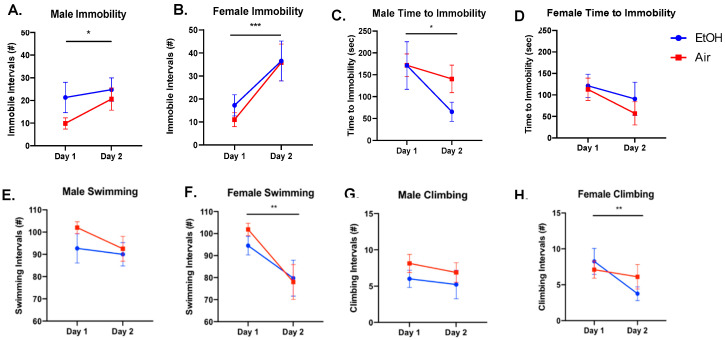
Frequency of immobility, climbing, swimming, and time to immobility during forced swim stress (FSS) sessions by adolescent air or ethanol (EtOH) exposure group on the first and second day of FSS sessions. Males showed a significant increase in immobile episodes (**A**) and decrease in time to immobility (**C**) on day two and no changes to climbing (**E**) or swimming behaviors (**G**). Females showed a significant increase in number of immobile episodes on day two (**B**), but no significant difference in time to immobility between days (**D**). Females also showed significant decreases in both climbing (**F**) and swimming behaviors on day two (**H**). There was no effect of ethanol exposure in either sex; a significant effect of day denoted as * *p* < 0.05, ** *p* < 0.01 and *** *p* < 0.001.

Following aCIE, adults that were assigned to the FSS condition were assessed for depressive-like behavior through scoring of number of immobile episodes and time to immobility. For number of immobile episodes, males showed a significant effect of day, with increased immobility from the first to the second day of FSS (Figure 3A; *F*(1,15) = 4.689, *p* = 0.047); however, there was no significant effect of ethanol exposure (*F*(1,15) = 1.411, *p* = 0.253) or a significant interaction (*F*(1,15) = 1.242, *p* = 0.283). The time to immobility was also measured and analyzed. Adolescent ethanol exposure did not significantly affect male time to immobility (*F*(1,18) = 0.818, *p* = 0.378). Males did show a significant effect of day, with a decrease in time to immobility on day two (*F*(1,18) = 0.5.835, *p* = 0.027), but no significant interaction (*F*(1,18) = 1.735, *p* = 0.204) (Figure 3C). For female number of immobile episodes, there again was a significant effect of day, showing increased immobility on day two (*F*(1,15) = 17.28, *p* < 0.001), but no significant effect of ethanol exposure (*F*(1,15) = 0.211, *p* = 0.652) and no significant interaction (*F*(1,15) = 0.282, *p* = 0.603) (Figure 3B). For time to immobility, females showed no significant effect of day (*F*(1,16) = 0.603, *p* = 0.449), no significant effect of ethanol exposure (*F*(1,16) = 1.781, *p* = 0.201), and no significant interaction (*F*(1,16) = 0.155, *p* = 0.699) (Figure 3D).

For the other forced swim behaviors, climbing and swimming, males showed no significant differences for ethanol exposure, day, or an interaction (Figure 3E,G; *p*-values > 0.05). Females did not show any significant effects for ethanol exposure (Figure 3F; climbing: *F*(1,15) = 0.180, *p* = 0.747; Figure 3H; swimming: *F*(1,15) = 0.172, *p* = 0.685), but did show a significant decrease in both climbing (*F*(1,15) = 8.980, *p* = 0.009) and swimming (*F*(1,15) = 12.37, *p* = 0.003) by day two. There were no significant interactions (climbing: *F*(1,15) =3.637, *p* = 0.076; swimming: *F*(1,15) = 0.692, *p* = 0.419).

### 3.3. Experiment 1: Social Interaction

**Figure 4:**
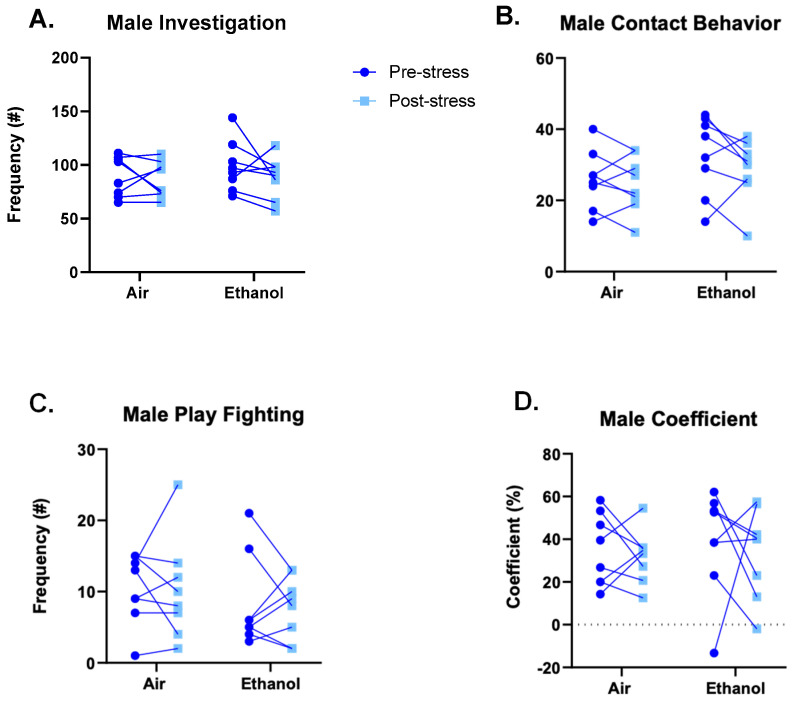
Frequencies of male social interaction behaviors, namely investigation (**A**), contact behaviors (**B**), play-fighting behaviors (**C**), and social preference/avoidance coefficient (**D**), compared between adolescent ethanol or air exposure and 24 h before and after two days of FSS. There were no significant effects of ethanol or stress for any measure in males.

We assessed social anxiety-like behavior as a within-subject design through social interaction with a novel partner twenty-four hours before and after two consecutive days of FSS. Males did not show any significant alteration in social behavior, including no significant differences in social investigation (Figure 4A; ethanol: *F*(1,14) = 0.389, *p* = 0.543; stress: *F*(1,14) = 1.500, *p* = 0.241; interaction: *F*(1,14) = 0.494, *p =* 0.494), contact behavior (Figure 4B; ethanol: *F*(1,14) = 1.687, *p* = 0.215; stress: *F*(1,14) = 1.985, *p* = 0.181; interaction: *F*(1,14) = 0.545, *p* = 0.473), play-fighting behavior (Figure 4C; ethanol: *F*(1,14) = 0.824, *p* = 0.379; stress: *F*(1,14) = 0.047, *p* = 0.831; interaction: *F*(1,14) = 0.017, *p* = 0.898), and preference/avoidance coefficient (Figure 4D; ethanol: *F*(1,14) = 0.229, *p* = 0.640; stress: *F*(1,14) = 0.321, *p* = 0.580; interaction: *F*(1,14) = 0.021, *p* = 0.888).

**Figure 5:**
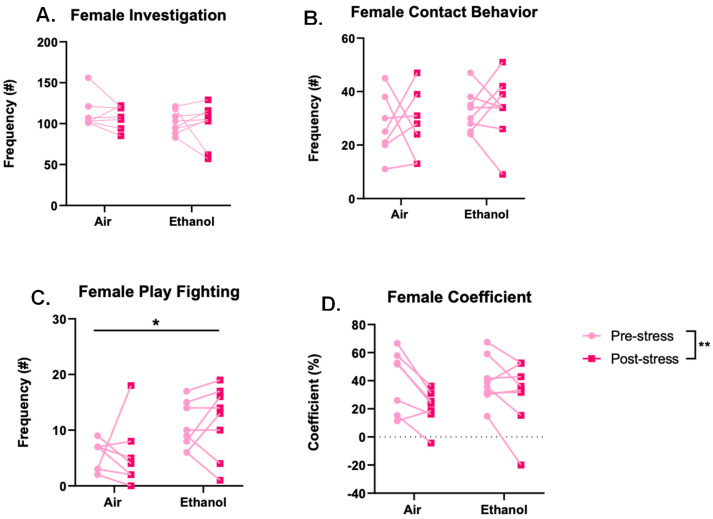
Frequencies of female social interaction behaviors, namely investigation (**A**), contact behaviors (**B**), play-fighting behaviors (**C**), and social preference/avoidance coefficient (**D**), compared between adolescent ethanol or air exposure and 24 h before and after two days of forced swim stress. Ethanol-exposed females showed increased play-fighting behavior, and females showed a lower preference/avoidance coefficient following stress. Significant main effects denoted as * *p* < 0.05 and ** *p* < 0.01.

Statistical analysis for identification of outliers (ROUT, Q = 1%) revealed one female outlier that was removed from all analyses. Females also did not show any significant effects for social investigation (Figure 5A; ethanol: *F*(1,13) = 1.648, *p* = 0.222; stress: *F*(1,13) = 0.521, *p* = 0.483; interaction: *F*(1,13) = 0.060, *p* = 0.810) or contact behavior (Figure 5B; ethanol: *F*(1,13) = 1.750, *p* = 0.209; stress: *F*(1,13) = 0.048, *p* = 0.829; interaction: *F*(1,13) = 0.001, *p* = 0.971). However, aCIE females showed a significant increase in play-fighting behavior compared to the air-exposed females (*F*(1,13) = 7.010, *p* = 0.020), but there was no significant difference in play-fighting behavior before and after stress (*F*(1,13) = 0.175, *p* = 0.683), and no significant interaction (*F*(1,13) = 0.105, *p* = 0.751) (Figure 5C). Finally, females showed a significantly lower coefficient after stress (*F*(1,13) = 12.95, *p* = 0.003), but there was no significant effect of ethanol (*F*(1,13) = 0.243, *p* = 0.630) and no significant interaction (*F*(1,13) = 1.558, *p* = 0.234) (Figure 5D).

### 3.4. Experiment 2: Light–Dark Box and Voluntary Ethanol Drinking

#### 3.4.1. Light-Dark Box

**Figure 6:**
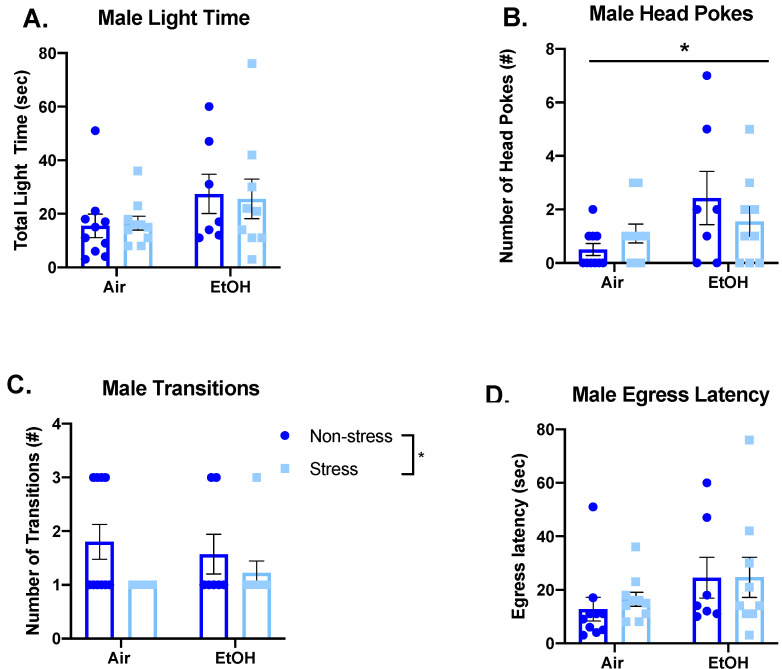
Comparison of male anxiety-like behaviors measured in the light–dark box by adolescent air or ethanol (EtOH) exposure in the non-stress group or following two days of forced swim stress, namely time spent in the light side (**A**), frequency of head pokes into the light side (**B**), number of transitions between the light and dark side (**C**), and latency to first cross into the dark side (**D**). Ethanol-exposed males showed a significant increase in number of head pokes, and stressed males showed a significant decrease in number of transitions. Significant main effects are denoted as * *p* < 0.05.

Following the aCIE and FSS sessions, general anxiety-like behavior in adulthood was assessed using the light–dark box. For males, this test did not reveal any significant effects for total time spent in the light side for either ethanol exposure (*F*(1,32) = 3.636, *p =* 0.066), or stress exposure (*F*(1,32) = 0.006, *p* = 0.937), and there was no significant interaction (*F*(1,32) = 0.068, *p* = 0.796) (Figure 6A). However, ethanol-exposed males showed a significant increase in head pokes compared to air-exposed males (*F*(1,32) = 5.050, *p* = 0.032), but there was no significant effect of stress on head pokes (*F*(1,32) = 0.066, *p* = 0.799) and no significant interaction (*F*(1,32) = 1.928, *p* = 0.175) (Figure 6B). Additionally, there was no significant effect of ethanol exposure (*F*(1,32) < 0.001, *p* = 0.990) and no significant interaction (*F*(1,32) = 0.759, *p* = 0.390) for male transitions, but when collapsed across ethanol exposure, stressed males showed a significant decrease in transitions (Mann–Whitney U = 113, sum of ranks = 303, 363, *p* = 0.037) (Figure 6C). Finally, there was no significant difference in egress latency among males for either ethanol exposure (*F*(1,32) = 3.146, *p* = 0.086) or stress exposure (*F*(1,32) = 0.114, *p* = 0.738), and there was no significant interaction (*F*(1,32) = 0.103, *p* = 0.751) (Figure 6D).

**Figure 7:**
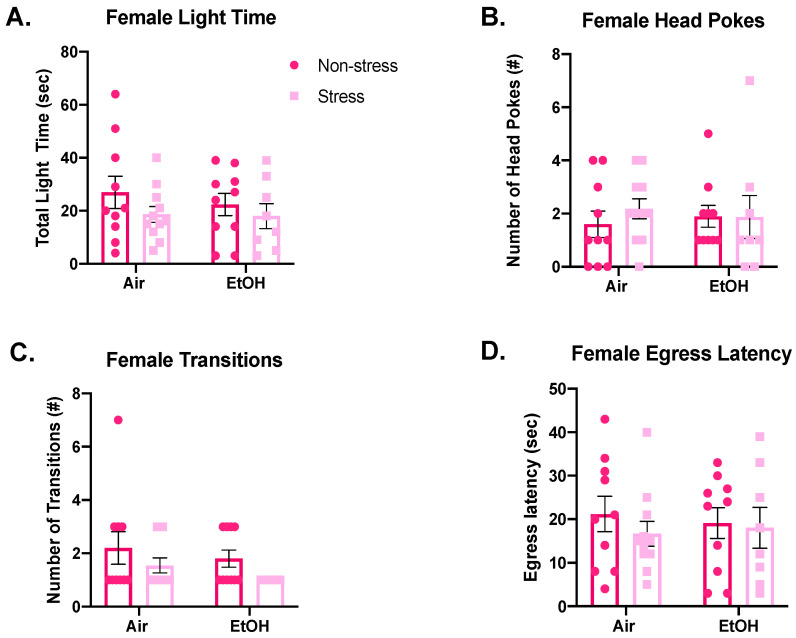
Comparison of female anxiety-like behaviors measured in the light–dark box by adolescent air or ethanol (EtOH) exposure in the non-stress group or following two days of forced swim stress, namely time spent in the light side (**A**), frequency of head pokes into the light side (**B**), number of transitions between the light and dark side (**C**), and latency to first cross into the dark side (**D**). There were no significant effects of ethanol or stress on any measure in females.

Similar to males, females did not show any significant differences among different exposure conditions for total time spent in the light side (Figure 7A; ethanol: *F*(1,35) = 0.3229, *p* = 0.574; stress: *F*(1,35) = 1.859, *p* = 0.182; interaction: *F*(1,35) = 0.185, *p* = 0.670). Additionally, there were no significant effects among females for number of head pokes (Figure 7B; ethanol: *F*(1,35) = 0.001, *p* = 0.995; stress: *F*(1,35) = 0.291, *p* = 0.593; interaction: *F*(1,35) = 0.345, *p* = 0.561), number of transitions (Figure 7C; ethanol: *F*(1,35) = 1.449, *p* = 0.237; stress: *F*(1,35) = 3.429, *p* = 0.073; interaction: *F*(1,35) = 0.034, *p* = 0.854), or egress latency (Figure 7D; ethanol: *F*(1,35) = 0.010, *p* = 0.923; stress: *F*(1,35) = 0.568, *p* = 0.456; interaction: *F*(1,35) = 0.213, *p* = 0.648).

#### 3.4.2. Voluntary Ethanol Drinking

**Figure 8:**
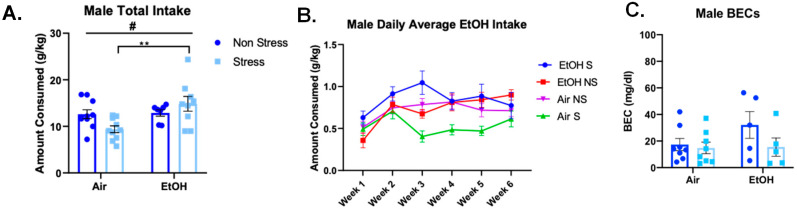
Male voluntary ethanol (10%) intake following FSS. (**A**) Cumulative intake across the eighteen sessions indicates a significant effect of ethanol exposure driven by the stressed groups. Ethanol-exposed males drank significantly more than air-exposed males, and stressed, ethanol-exposed males consumed significantly more than stressed, air-exposed males. Significant effect of ethanol denoted as # *p* < 0.05; interactions denoted as ** *p* < 0.01. (**B**) Daily intake averages per week indicates significant ethanol × stress × week interactions. (**C**) BECs at the end of the final drinking session.

To identify differences in voluntary ethanol consumption, animals underwent a total of eighteen drinking sessions over six weeks. For cumulative total consumption across the six weeks, ethanol-exposed males showed significantly higher total ethanol consumption, compared to air-exposed males (*F*(1,32) = 7.064, *p* = 0.012) and a significant interaction (*F*(1,32) = 5.739, *p* = 0.023), but no significant effect of stress alone (*F*(1,32) = 0.348, *p =* 0.560) (Figure 8A). Follow-up with Sidak’s post-hoc test revealed that stressed, aCIE males consumed significantly more ethanol than stressed, air-exposed males (*p* = 0.005).

After averaging daily intake per week, there was a significant effect of week in males (Figure 8B; *F*(3.9,123.5) = 8.137, *p* < 0.0001). Additionally, aCIE males consumed significantly more than air-exposed males across the six-weeks (*F*(1,32) = 6.956, *p* = 0.013) and males showed a significant ethanol exposure and stress exposure interaction (*F*(1,32) = 5.872, *p* = 0.021) and a significant three-way interaction between ethanol exposure, stress exposure, and week (*F*(5,160) = 3.160, *p* < 0.01). However, a post-hoc Sidak’s test failed to find significant differences between the groups at any week. BECs were also measured for all animals following the last session of voluntary ethanol drinking. Males did not show any significant differences in BECs (ethanol: *F*(1,22) = 1.548, *p* = 0.227; stress: *F*(1,22) = 2.384, *p* = 0.137; interaction: *F*(1,22) = 1.303, *p* = 0.266) (Figure 8C).

**Figure 9:**
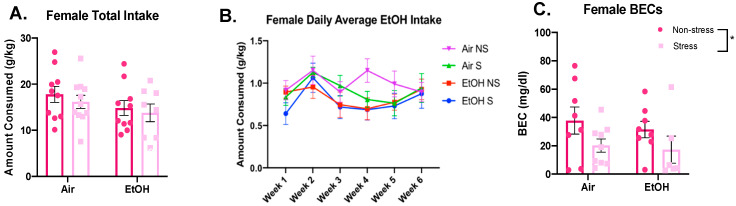
Female voluntary ethanol (10%) intake following FSS. Cumulative intake across the eighteen drinking sessions (**A**) and intake averages for each week (**B**) are shown alongside BECs taken immediately after the final drinking session (**C**). No significant effects of ethanol exposure or stress for either intake measure. Significant stress effect in BECs denoted as * *p* < 0.05.

For female cumulative total consumption, there were no significant differences for adolescent ethanol exposure (*F*(1,35) = 2.574, *p* = 0.118) or stress exposure (*F*(1,35) = 0.634, *p* = 0.431) and no significant interaction (*F*(1,35) = 0.030, *p* = 0.864) (Figure 9A). For daily averages per week, females also showed a significant effect of week (Figure 9B; *F*(3.4,117.9) = 4.074, *p* = 0.006), but no significant effect of ethanol (*F*(1,35) = 2.337, *p* = 0.135) or stress (*F*(1,35) = 0.536, *p* = 0.469), and no significant interactions (week x ethanol: *F*(5,175) = 0.829, *p* = 0.531; week × stress: *F*(5,175) = 1.022, *p* = 0.406; ethanol x stress: *F*(1,35) = 0.059, *p* = 0.810; week x ethanol x stress: *F*(5,175) = 0.992, *p* = 0.424). For BECs, stressed females showed a significant decrease in BECs compared to non-stressed females (*F*(1,27) = 4.602, *p* = 0.041), but did not show any significant differences for aCIE animals (*F*(1,27) = 0.387, *p* = 0.539), and no significant interaction (*F*(1,27) = 0.052, *p* = 0.821) (Figure 9C).

## 4. Discussion

The present study examined the effects of moderate adolescent ethanol exposure on adult anxiety-like and voluntary ethanol drinking behaviors to identify potential differences in sensitivity to acute stress in adulthood resulting from ethanol exposure. Generally, our hypothesis was that aCIE would heighten sensitivity to adult FSS for both anxiety-like behavior and ethanol intake, with some expected sex-specific effects. Consistent with our hypothesis, aCIE followed by FSS in adulthood increased voluntary ethanol intake only in males. Surprisingly, females did not show any significant differences between groups for voluntary ethanol intake following aCIE or stress exposure. We also did not find many significant effects of aCIE or stress exposure in either males or females for general and social anxiety-like behavior, except for an increase in play-fighting behaviors in aCIE females and a decrease in female social preference after stress. The little change in these behaviors indicate that this moderate ethanol exposure may not produce long-term alterations of these behaviors, as observed with higher-level ethanol exposures. However, there are several methodological factors that should be considered, as discussed below.

Given the use of FSS as our stress exposure, we examined differences in immobility during the forced swim sessions. In contrast to previous findings that have demonstrated that males show higher levels of immobility in the forced swim test following adolescent ethanol exposure (vapor, 14 h per day for thirty-five days, BECs ~163 mg/dL) [47], we did not find any significant differences in time to immobility or number of immobile episodes in aCIE males or females. These differences are likely due to the differences in ethanol exposure intensity (i.e., higher BECs, a longer exposure period, and/or more frequent exposures). Other procedural differences may also have contributed, such as their model examined behaviors during a five-minute test on the second exposure day, as well as tested animals following other behavioral assays, including acoustic startle response and the open-field conflict test. Importantly, though, we did see a significant effect of day in both males and females, indicating a possible adaptive response or increased depression-like behavior, even by the second exposure.

In looking at the effects observed in the social interaction paradigm, we did not identify an increase in social anxiety-like behavior in males, as had been previously reported following adolescent ethanol exposure with restraint stress in adulthood [17,26]. We did, however, identify an increase in female play-fighting behavior following aCIE, indicating higher levels of a more adolescent-typical social activity in aCIE adult females, and a decrease in female social preference coefficient following FSS, indicating higher levels of social anxiety-like behavior following stress exposure, which have not been previously shown. Although these effects were not interactions between aCIE and stress, the timing between stress exposure and testing for social interaction likely influence the social behaviors, as Varlinskaya, Kim [26] reported alterations both on the last day of stress exposure and one week following restraint stress exposure, while in the current study we only tested animals twenty-four hours after FSS. Another element that may have influenced the findings from our assessment of social behavior is the within-subject design for this specific experiment. Many studies use a between-subject design in assessment of these behaviors; however, since this assay allows for the use of a within-subject design with a novel partner, that is how we elected to design this experiment in order to observe stress-induced differences in consideration of individual variability. Nevertheless, our lab recently showed that social behavior in ethanol naïve adults was unaffected by FSS [45], an effect that we replicated in the current study. Importantly, our findings suggest that moderate aCIE exposure was not sufficient to increase sensitivity to this stress exposure.

When general anxiety-like behavior was assessed using the light–dark box, we expected that aCIE would increase sensitivity to the anxiogenic effects of FSS. Thus, it was surprising to see no effects of aCIE or stress alone, nor alcohol-stress interactions with this assay. These results contrast previous work that has shown either increased [13,14,16,48] or decreased [21,22,49] anxiety-like behavior in adult males following exposures to much higher levels of ethanol during adolescence, which in and of itself suggests that there are many factors that influence the alterations in anxiety-like behavior following adolescent ethanol exposure. In the current study, a component that may have influenced the lack of anxiogenic effect is that our testing conditions may have been too anxiogenic, resulting in a floor effect. Our data showed that even control animals spent little time exploring the light side (<10%), and the majority of animals did not return to explore the light side after their initial transition into the dark side. This is not uncommon; a recent study by Varlinskaya and colleagues examining adolescent ethanol exposure showed a similar floor effect using the light–dark box [50]. Others have identified that lighting of the testing room plays a significant role in the light-side time exploration using this task [51,52,53], and this may be a contributing factor to the floor effects seen in this study. Additionally, for females that on average spent more time in the light side compared to males, previous work has shown that estrous cycle may play a role in measures of anxiety-like behavior in other assays of anxiety-like behavior, such as the elevated plus-maze [54]. These effects have not been extensively examined for the light–dark box measure specifically but could also have played a role in the observed results, as females, regardless of condition, showed high variability of time spent in the light side. Despite these potential factors that may have influenced the expression of anxiogenic behaviors, we also did not observe an anxiolytic effect of aCIE, as those that have been previously reported [21,22], albeit there was a trend (*p* = 0.066) toward increased time in the light side of aCIE males relative to controls, independent of stress exposure. However, increased anxiety-like behavior was apparent 24 h into withdrawal from as little as 3 days of intermittent ethanol vapor exposure during a presumptive adolescent period (based on animal weights) [55], suggesting that the timing of assessment is another important factor to consider. Thus, the current findings may be somewhat inconclusive regarding the effects of aCIE and interactions with adult FSS on anxiety-like behaviors in the light–dark box given our experimental conditions and timing of assessment. Specifically, it will be important to determine whether aCIE produces alterations in anxiety-like behavior during acute withdrawal (i.e., 24 h into withdrawal), as well as employing other assays that may better probe behavioral alterations and tracking estrous cycle to see how this may influence anxiety-like behavior.

An interesting finding from this study was the sex-dependent effects of the aCIE and stress exposures on adult voluntary ethanol drinking. Our aCIE vapor exposure increased adult male voluntary ethanol intake, consistent with some previous findings [15,16]. Additionally, we observed an interesting interaction between ethanol exposure and stress exposure where stress increased drinking in the aCIE males, but blunted drinking in air-exposed males. These findings indicate that aCIE exposure impairs adult stress-reactive reductions in ethanol drinking in males. It is interesting to note that this moderate aCIE exposure did produce increased drinking in males in the long term, while previous work has shown no difference in adult voluntary consumption following higher-level adolescent exposure [56,57]. One potential difference is that many studies order animals from vendors that are shipped during the vulnerable early-life period, and there is compelling evidence that this shipment stress may alter future behaviors [58]. The animals used in the present study were bred in house and therefore bypassed the shipment stress during early adolescence, potentially altering the way the ethanol exposure impacts these behaviors later on. Additionally, the study by Gass and colleagues used a model that produced much higher BECs in Long Evans rats during adolescence and did not observe a change in ethanol intake in adulthood when using operant self-administration [21], whereas we used Sprague–Dawley rats and a voluntary single bottle ethanol intake test in adulthood. Taken together, it is clear that regardless of level of ethanol exposure during adolescence and the impact of adult stress, the long-term effects of these exposures on adult ethanol intake are not consistent and more studies are required to understand the various interactions that may exist.

In contrast, females did not show any differences in voluntary ethanol intake, which was surprising because we expected the females to be more vulnerable to stress-reactive drinking based on previous literature that has shown increases in female intake in response to both pharmacological and predator odor stressors [35,42,59]. Additionally, Fullgrabe and colleagues reported increased ethanol intake in females following the combination of adolescent ethanol and stress exposures [42]. Despite some of these reports and consistent with our findings, it has been suggested that females are less vulnerable to the long-term effects of these exposures when examining other behaviors in adulthood, such as depression-like and social anxiety-like behaviors [26]. Importantly, females did show relatively higher intake compared to males, which has been consistently shown throughout the literature [60,61], so it may be possible that the thirty minutes of access with our model was not enough time to really observe a true escalation in intake for the females and we may be seeing a ceiling effect under our testing conditions. However, we still did not see the reduction in drinking in the air-stressed females that we saw in the males, so this effect does appear to be sex dependent. It was interesting that we observed a decrease in BECs of stressed females following the final drinking session, despite no difference in actual ethanol intake of these animals. It is unclear why this effect was observed but may indicate a difference in ethanol metabolism in these animals. Future research should follow up on this effect.

These findings suggest that the mechanisms underlying long-lasting changes to various behaviors are likely disrupted in a sex- and exposure intensity-dependent way. While the moderate-level exposure used in this study is sufficient to produce disruptions in the mechanisms underlying male drinking patterns, females show resistance to these disruptions. Therefore, this moderate exposure may not impact female drinking in the long term in the way that other higher-intensity adolescent ethanol exposures do. Taken together, adolescent males appear to be more vulnerable to the effects of moderate adolescent ethanol on long-term drinking patterns, both generally and in response to stress exposure, while adult females show resistance to the long-term behavioral consequences of moderate ethanol exposure during adolescence, even in combination with adult stress exposure.

Another important factor to consider with our aCIE paradigm is that the BECs showed a significant decrease from the first to the last exposure, suggesting that animals did not maintain the same intoxication levels by the end of the exposure period when ethanol vapor concentrations remained the same. Interestingly, this phenomenon has also been reported using intragastric administration of ethanol during adolescence [38]. This may be due to many different factors, including metabolic tolerance or an age effect between the first and last exposure [62,63]. This requires further examination of these different factors in their contribution to the decreased BECs in a vapor exposure model and may be an important consideration for future studies in deciding if this is an appropriate model for their specific study. Nevertheless, even these moderate and gradually decreasing BEC levels over a brief period in adolescence were sufficient to interact with the stress exposure to produce several long-term effects in adulthood.

The final ethanol exposure-related considerations are the age and duration of exposure, which have become important factors in modeling exposures to various substances in adolescents, particularly ethanol. Findings suggest that exposures during early adolescence (P25-45) may have more detrimental effects on certain behaviors, such as increased adult ethanol intake and both general and social anxiety-like behaviors, than when exposure occurs during late adolescence (P45-65), especially in males [15,17,50]. While our exposure occurred in the early adolescent window, our model did not span the entire twenty days, and therefore, may be missing a critical vulnerable point in these animals that could contribute to the lack of effects on anxiety-related behaviors. Interestingly, though, previous work has shown that even an abbreviated window of exposure (P34-38) does still produce long-term decreases in anxiety; however, this was shown following much higher levels of ethanol exposure, with BECs ~172 mg/dL [22]. Therefore, the interaction between ethanol intoxication level and window of adolescent exposure may be a crucial factor that should be addressed in follow-up studies to fully examine the critical point for this type of exposure in adolescence.

It is worth noting that there were also some general limitations to this study. First, animals were rehoused into larger groups during the adolescent ethanol exposure, and then separated back into original housing configurations of two to three per cage after the exposure was complete. However, even with just the two instances of rehousing required, this social instability may have potentially caused some additional stress to the animals (as reviewed by [64]) and should be taken into consideration with these results and the extant literature, given that the focus of this study is on the vulnerability of this adolescent period. Additionally, in some of the experiments, most groups contained two to three littermates which may have inflated significance in some cases [65]. However, we also analyzed the data by averaging results by litter and found the same effects as when analyzing by animal (data not shown), suggesting that litter effects did not contribute to the observed findings.

## 5. Conclusions

In conclusion, with an alarming proportion of adolescents consuming alcohol [3], it is imperative to identify the immediate and long-term consequences that may result. Continued research is a necessity in further identifying the ways alcohol consumption during this highly vulnerable adolescent period affects an individual in the long term, especially in regard to the stress response system, and at what levels of intoxication these life-long consequences may emerge. As of 2018, 14.4 million adults had AUD [3], and 19.1% of U.S. adults are affected by an anxiety disorder every year [66]. Identifying a relationship between different life factors, such as adolescent alcohol consumption and stress, and the ways in which they interact and contribute to these staggering numbers is crucial. The present study identified effects of a moderate exposure to ethanol during adolescence and interactions with exposure to stress in adulthood, particularly related to ethanol intake. While these findings may suggest that there may not be as many extreme and long-lasting alterations at these lower levels of intoxication to the stress response system that would manifest as significant effects on adult anxiety-like behaviors following exposure to stress, a heightened propensity to consume ethanol following acute stress is a major effect that can have life-long implications. Importantly, many of the brain networks involved in emotion regulation and reward are still developing and maturing during adolescence [67,68,69] suggesting that even moderate exposure to ethanol may disrupt this developmental trajectory. In conclusion, results such as these can aid us in putting together a clearer picture of the deficits and alterations that may be produced from these exposures, and this information can be used to identify ways in which long-term consequences, such as AUD and anxiety disorders, can be prevented or rescued.
